# Notch2 is required in somatic cells for breakdown of ovarian germ-cell nests and formation of primordial follicles

**DOI:** 10.1186/1741-7007-11-13

**Published:** 2013-02-13

**Authors:** Jingxia Xu, Thomas Gridley

**Affiliations:** 1 The Jackson Laboratory, Bar Harbor, Maine 04609. USA; 2 Department of Molecular and Biomedical Sciences, University of Maine, Orono. ME 04469. USA; 3 Center for Molecular Medicine, Maine Medical Center Research Institute, Scarborough. ME 04074. USA

**Keywords:** oogenesis, Notch signaling, apoptosis.

## Abstract

**Background:**

In the mouse ovary, oocytes initially develop in clusters termed germ-cell nests. Shortly after birth, these germ-cell nests break apart, and the oocytes individually become surrounded by somatic granulosa cells to form primordial follicles. Notch signaling plays essential roles during oogenesis in *Drosophila*, and recent studies have suggested that Notch signaling also plays an essential role during oogenesis and ovary development in mammals. However, no *in vivo *loss-of-function studies have been performed to establish whether Notch family receptors have an essential physiological role during normal ovarian development in mutant mice.

**Results:**

Female mice with conditional deletion of the *Notch2 *gene in somatic granulosa cells of the ovary exhibited reduced fertility, accompanied by the formation of multi-oocyte follicles, which became hemorrhagic by 7 weeks of age. Formation of multi-oocyte follicles resulted from defects in breakdown of the primordial germ-cell nests. The ovaries of the *Notch2 *conditional mutant mice had increased numbers of oocytes, but decreased numbers of primordial follicles. Oocyte numbers in the *Notch2 *conditional mutants were increased not by excess or extended cellular proliferation, but as a result of decreased oocyte apoptosis.

**Conclusions:**

Our work demonstrates that *Notch2*-mediated signaling in the somatic-cell lineage of the mouse ovary regulates oocyte apoptosis non-cell autonomously, and is essential for regulating breakdown of germ-cell nests and formation of primordial follicles. This model provides a new resource for studying the developmental and physiological roles of Notch signaling during mammalian reproductive biology.

## Background

Primordial germ cells in mice arise in the proximal extraembryonic mesoderm of the mouse embryo, and migrate to the embryonic gonad primordia [1,2]. In female mice, primordial germ cells that enter the embryonic ovaries divide mitotically until approximately embryonic day (E) 13.5. These mitotic oocyte progenitors are termed oogonia. Oogonia enter meiosis after E13.5, and are then termed oocytes. Oocytes arrest in the diplotene stage of the first meiotic division.

Initially, oocytes develop in clusters termed germ-cell nests (also called germ-cell clusters, cysts, or syncytia). These nests arise through the processes of incomplete cytokinesis and cellular aggregation [2-6]. During the first few days after birth, the germ-cell nests break apart, and the oocytes individually become surrounded by somatic cells to form primordial follicles. Temporally, the process of germ-cell nest breakdown and primordial-follicle formation is accompanied by the apoptotic cell death of approximately two-thirds of the oocytes. The surviving oocytes become surrounded by a single layer of somatic pre-granulosa cells, forming the primordial follicles [2-5]. Primary follicles are formed from the primordial follicles as the oocytes start to grow and the surrounding somatic pre-granulosa cells become cuboidal and proliferative. The prevailing view in the field has been that the oocytes which are present in the primordial follicles of the ovaries represent the entire reservoir of gametes available to a female mouse throughout its reproductive life. However, a vigorous debate has developed over the existence of female germ-line stem cells in ovaries of mice and humans [7,8].

Breakdown of germ-cell nests and formation of primordial follicles are key early events in mammalian folliculogenesis. Breakdown of germ-cell nests occurs during the same time window as the apoptotic death of approximately two-thirds of the oocytes within those nests; however, the mechanistic connection between these two events is not clear. It has long been known that exposure of neonatal mice to various estrogenic compounds results in formation of multi-oocyte follicles, and it is believed that defects in the process of germ-cell nest breakdown leads to the formation of these multi-oocyte follicles [4,5].

The Notch signaling pathway is an evolutionarily conserved, intercellular signaling mechanism [9,10]. Notch signaling frequently plays a crucial role in precursor cells, making binary cell-fate decisions. However, Notch signaling also regulates additional developmental decisions, such as boundary formation between cell populations, cell proliferation, and cell death. Notch family receptors are large single-pass Type I transmembrane proteins. In mammals, four Notch family receptors have been described, encoded by the *Notch1*, *2*, *3 *and *4 *genes.

A Notch family receptor exists at the cell surface as a proteolytically cleaved, non-covalently associated heterodimer, consisting of a large ectodomain and a membrane-tethered intracellular domain. During canonical Notch signaling, Notch receptors interact with ligands that are also single-pass Type I transmembrane proteins. This restricts the Notch pathway to regulating juxtacrine intercellular interactions. In mammals, the canonical Notch ligands are encoded by the Jagged (*Jag1*, *Jag2*) and Delta-like (*Dll1*, *Dll3*, *Dll4*) gene families.

The signal induced by ligand binding is transmitted intracellularly by a process involving proteolytic cleavage of the receptor and nuclear translocation of the intracellular domain of the Notch family protein. The receptor/ligand interaction induces two additional proteolytic cleavages in the membrane-tethered fragment of the Notch heterodimer. The final cleavage, catalyzed by the gamma-secretase complex, frees the intracellular domain of the Notch receptor from the cell membrane. The cleaved fragment translocates to the nucleus owing to the presence of nuclear localization signals located in the Notch intracellular domain. Once in the nucleus, the Notch intracellular domain forms a complex with a sequence-specific DNA binding protein, the RBPJ protein, (also known in mammals as CSL or CBF1), and activates transcription of Notch target genes.

Notch signaling plays an essential role during oogenesis in *Drosophila*, and is required at several different stages of oocyte development [11-13]. Recent work has suggested that Notch signaling probably also plays an essential role during oogenesis and ovary development in mammals. The Notch2 receptor is expressed at high levels in pre-granulosa [14,15] and granulosa [16] cells of the neonatal and adult mouse ovary, and *ex vivo *culture of neonatal mouse ovaries in gamma-secretase inhibitors (which abrogate Notch signaling) resulted in defects in granulosa-cell proliferation and primordial-follicle formation [14,15]. However, no *in vivo *loss-of-function studies have been performed to establish whether Notch family receptors have an essential physiological role during normal ovary development. Mice homozygous for a *Notch2 *null allele die early during embryogenesis [17,18], thus necessitating a conditional gene-deletion strategy to examine the requirement for *Notch2 *gene function during oogenesis.

In this paper, we report that *Notch2 *gene function in the somatic-cell lineage of the mouse ovary is essential for breakdown of germ-cell nests and formation of primordial follicles, and that *Notch2 *function in granulosa cells non-cell-autonomously regulates apoptosis of oocytes in the early postnatal period.

## Results

### Female *Amhr2-Cre/+;Notch2^flox/- ^*mice exhibit reduced fertility

**Figure 1:**
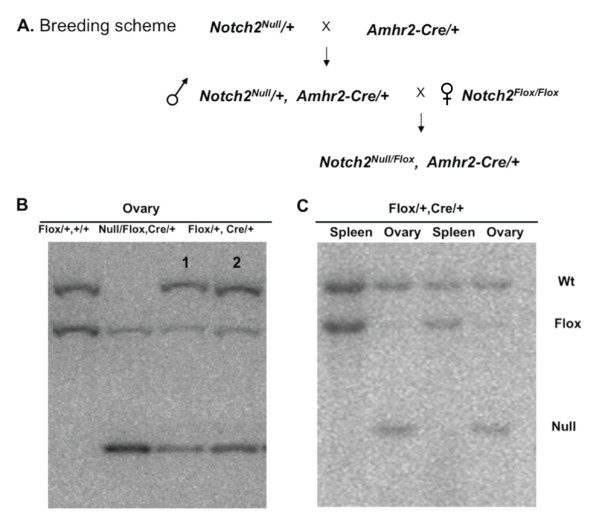
**Generation of mice with ovary-specific *Notch2 *gene deletion**. (**A**) Breeding scheme to generate mice with ovary-specific *Notch2 *gene deletion using the *Amhr2-Cre *driver line. (**B**, **C**) Efficiency and specificity of recombination of the *Notch2^flox ^*allele by the *Amhr2-Cre *driver. (**B) **Southern-blot analysis of genomic DNA from ovaries obtained from: (lane 1) *Notch2^flox^*/+;+/+ control (mouse has no *Cre *driver); (lane 2) *Notch2^flox^*/*Notch2^null^*;*Amhr2*-*Cre*/+ mutant; (lanes 3 and 4), two individual *Notch2^flox^*/+;*Amhr2*-*Cre*/+ mice (heterozygous for the *Notch2^flox ^*allele). All mice were four to five weeks old. (**C**) Southern blot of ovary and spleen DNA to test the specificity and efficiency of *Amhr2-Cre*-mediated recombination. DNA was isolated from two individual *Notch2^flox^*/+;*Amhr2*-*Cre*/+ control mice (heterozygous for the *Notch2^flox ^*allele) at 9 weeks of age. There was almost complete deletion of the *Notch2^flox ^*allele in ovary DNA, but not in spleen DNA. Note that the band resulting from Cre-mediated recombination (labeled 'Null') was present only in *Amhr2*-*Cre*/+ ovaries.

The *Notch2 *gene is expressed at high levels in granulosa cells, the somatic-cell lineage of the ovarian follicle. To assess whether *Notch2 *gene function is required for correct development of the mouse ovary, we conditionally deleted the *Notch2 *gene in the somatic lineages of the mouse ovary using the anti-Müllerian hormone Type II receptor- Cre recombinase (*Amhr2-Cre*) driver line [19]. Using Southern blotting analysis, we found efficient excision of the *Notch2^flox ^*allele by the *Amhr2-Cre *line in ovary DNA, but not in spleen DNA (Figure 1). Female *Amhr2-Cre/+;Notch2^flox/- ^*and control littermate mice were mated to wild-type C57BL/6J male mice over a 6-month period, then we assessed the fertility and reproductive performance of both groups of mice. The *Amhr2-Cre/+;Notch2^flox/- ^*mice exhibited reduced fertility compared with the littermate controls. Female *Amhr2-Cre/+;Notch2^flox/- ^*mice had only approximately one-third as many progeny as control littermates over the 6-month period, even though *Amhr2-Cre/+;Notch2^flox/- ^*mice had similar total litter numbers to the controls (Table 1).

**Table 1:** Six-month mating data for *Amhr2-Cre/+;Notch2^flox/- ^*and control littermate female mice.

Genotype	Mice, n	Average total progeny^a^	Average litter size^a^	Average total litters^a^
*+/+;Notch2^flox^*/+	7	60.7 ± 3.5	9.7 ± 0.7	6.29 ± 0.5
*Amhr2-Cre/+;Notch2^flox^*/+	8	61.6 ± 6.4	9.9 ± 0.6	6.25 ± 0.5
*Amhr2-Cre/+;Notch2^flox/- ^*	10	22.3 ± 7.1^a^	3.9 ± 1.1^b^	5.75 ± 1.0

^a^Values are expressed as mean ± SD. ^b^*P *< 0.001.

### *Notch2 *gene deletion in granulosa cells leads to formation of multi-oocyte follicles and persistence of germ-cell nests

**Figure 2:**
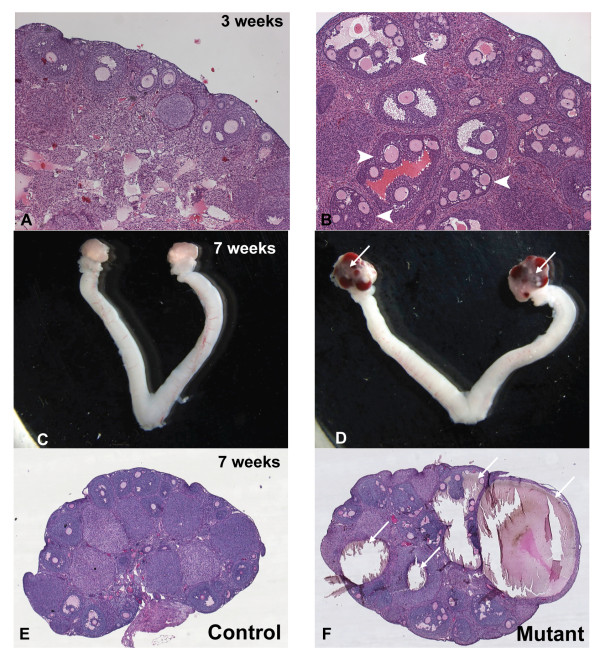
**Ovaries of *Amhr2-Cre/+;Notch2^flox/- ^*mice contain multi-oocyte follicles that become hemorrhagic**. (**A**, **C**, **E**) Littermate controls; (**B**, **D**, **F**) *Amhr2-Cre/+;Notch2^flox/- ^*mutants. (**A**, **B**) Multi-oocyte follicles were present in 3-week-old *Amhr2-Cre/+;Notch2^flox/- ^*female mice (**B**. arrowheads). (**C**-**F**) Follicles in *Amhr2-Cre/+;Notch2^flox/- ^*females became hemorrhagic by approximately 7 weeks of age (**D**, **F**, arrows).

Histological analysis of ovaries taken from *Amhr2-Cre/+;Notch2^flox/- ^*mice at 3 weeks of age identified the presence of multi-oocyte follicles (Figure 2). This phenotype was completely penetrant, as all *Amhr2-Cre/+;Notch2^flox/- ^*mice developed multi-oocyte follicles. As many as 10 oocytes were present in a single follicle in the *Amhr2-Cre/+;Notch2^flox/- ^*ovaries. By 7 weeks of age, the follicles in the *Amhr2-Cre/+;Notch2^flox/- ^*mutant ovaries became hemorrhagic (Figure 2D, F).

**Figure 3:**
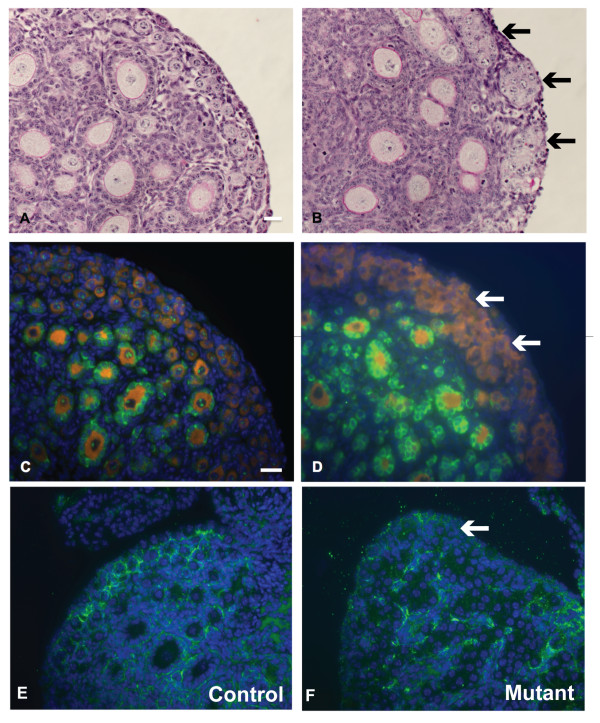
**Germ-cell nests persisted in *Amhr2-Cre/+;Notch2^flox/- ^*mice**. (**A**, **B**) Germ-cell nests in (**A**) littermate control and (**B**) 8-day-old *Amhr2-Cre/+;Notch2^flox/- ^*mouse. (**B**) In the mutant mouse, the germ-cell nests were still visible (black arrows), whereas in (**A**) the nests had broken down and the oocytes were assembled into primordial follicles. Slides were stained using the Periodic-Acid-Schiff (PAS) (**C**) By postnatal day (PND)3, most oocytes expressing the cytoplasmic germ-cell marker Vasa (orange) in the cortical region of the ovary had been recruited into primordial follicles in the control littermate mice. (**D**) However, Vasa-positive germ cells in the cortical region of the *Amhr2-Cre/+;Notch2^flox/- ^*mice were still in germ-cell nests (white arrows) at PND3. Cuboidal granulosa cells of primary follicles in the medulla of both littermate and mutant ovaries expressed anti-Müllerian hormone (AMH; green). Cell nuclei were counterstained with 4',6-diamidino-2-phenylindole (DAPI) (blue). (**E**) Laminin immunostaining (green) showed that by PND3, primordial follicles in the cortex of the ovary of control littermate mice were surrounded by a layer of basement membrane. (**F**) However, in *Amhr2-Cre/+;Notch2^flox/- ^*mice ovaries at the same stage, the oocytes remained in germ-cell cysts and were not individually surrounded by a basement membrane. Scale bar: (**A**, **B**): 20 μm; (**C**-**F**): 50 μm.

The presence of multi-oocyte follicles in *Amhr2-Cre/+;Notch2^flox/- ^*mice suggests that *Notch2*-mediated signaling may be required for breakdown of the primordial germ-cell nests. In wild-type mice, breakdown of germ-cell nests and formation of primordial follicles are normally complete within a few days of birth. Histological analyses on ovaries isolated at postnatal day (PND) 8 from *Amhr2-Cre/+;Notch2^flox/- ^*and control littermate mice showed that, whereas virtually all germ-cell nests in the control mice had broken down to form primordial follicles, there was persistence of germ-cell nests in the cortex of the *Amhr2-Cre/+;Notch2^flox/- ^*ovaries (Figure 3B). This was confirmed by immunofluorescence staining of ovaries isolated at PND3, using an antibody to the cytoplasmic germ-cell marker Vasa (*Ddx4*). In littermate control ovaries at PND3, most Vasa-positive cells had already been assembled into individual primordial follicles (Figure 3C). However, in the *Amhr2-Cre/+;Notch2^flox/- ^*ovaries, most Vasa-positive cells, particularly near the cortical surface of the ovary, remained in germ-cell nests (Figure 3D). By PND3, laminin immunostaining showed that in control littermate mice, the primordial follicles in the cortex of the ovary were surrounded by a basement membrane (Figure 3E), whereas in *Amhr2-Cre/+;Notch2^flox/- ^*ovaries, the oocytes remained in the germ-cell nests and were not individually surrounded by a basement membrane (Figure 3F). These data indicate that *Notch2 *gene function in pre-granulosa cells is required for breakdown of the primordial germ-cell nests and assembly of primordial follicles.

### Ovaries of *Amhr2-Cre/+;Notch2^flox/- ^*mice have increased numbers of oocytes but decreased numbers of primordial follicles

**Figure 4:**
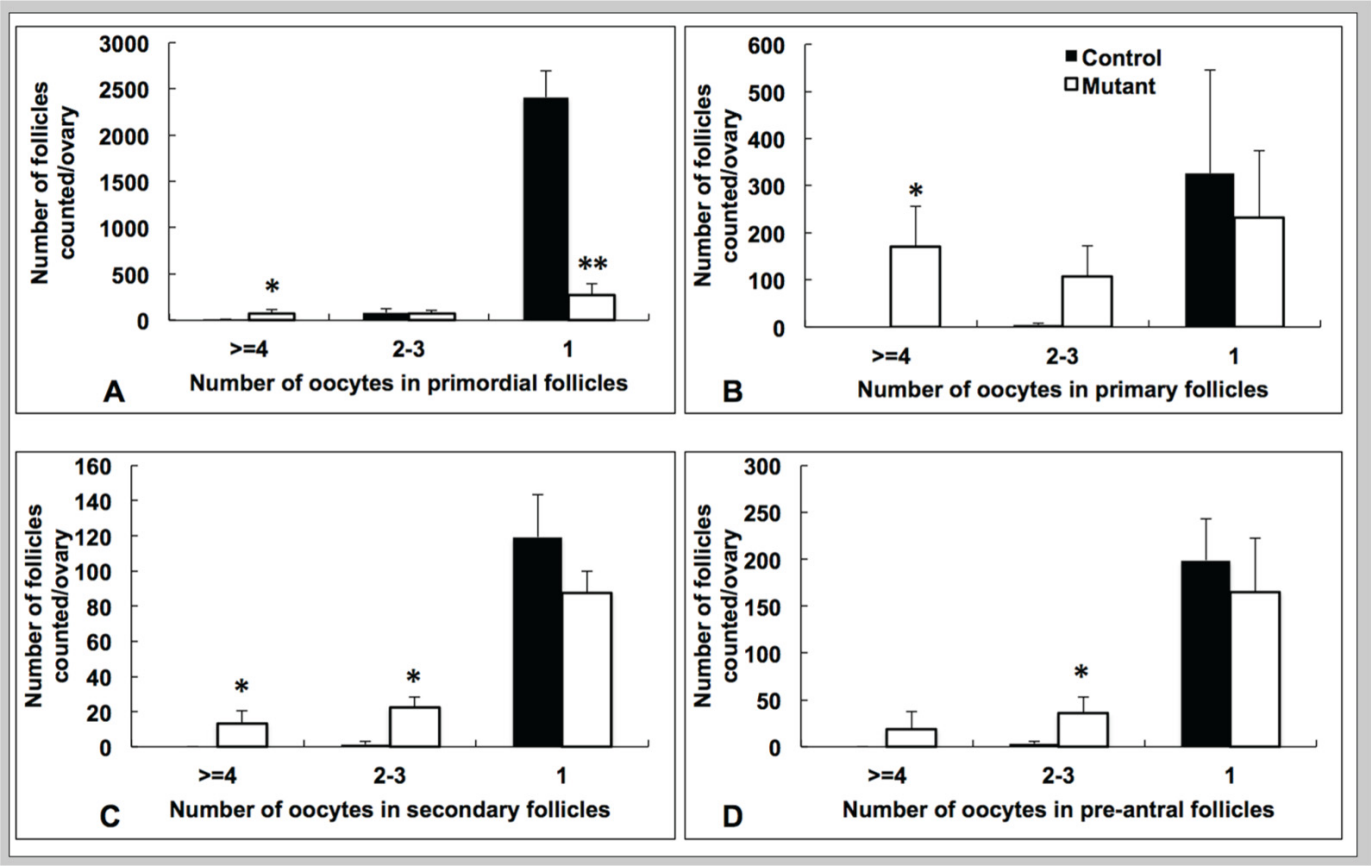
**Assessment and quantification of follicle types in the ovaries of prepubertal *Amhr2-Cre/+;Notch2^flox/- ^*and control littermate mice**. (**A**-**D**) Follicle types were assessed and counted in serial sections of ovaries isolated from *Amhr2-Cre/+;Notch2^flox/- ^*(white bars) and control littermate (black bars) mice at postnatal day 18. (**A**-**C**) *Amhr2-Cre/+;Notch2^flox/- ^*ovaries exhibited a significant increase in the number of multi-oocyte follicles (containing four or more oocytes) of (**A**) the primordial, (**B**) primary and (**C**) secondary follicle types compared with littermate control ovaries. *Amhr2-Cre/+;Notch2^flox/- ^*ovaries also exhibited a significantly reduced number of primordial follicles containing a single oocyte. * *P *< 0.05; ** *P *< 0.005.

To quantify oocyte numbers in *Amhr2-Cre/+;Notch2^flox/- ^*and control littermate mice, we counted serial sections of ovaries isolated at PND18. This time point was chosen because it is a point shortly before the female mouse reaches sexual maturity. *Amhr2-Cre/+;Notch2^flox/- ^*mice had approximately 20% more oocytes than ovaries from littermate controls (Table 2). We then quantified the type of follicle present (that is, primordial, primary, secondary, pre-antral, and antral) and assessed whether these follicles contained a single oocyte, two or three oocytes, or four or more oocytes (Table 3, Figure 4). For all follicle types, only the *Amhr2-Cre/+;Notch2^flox/- ^*mice had four or more oocytes per follicle present in the ovaries (Figure 4). We divided the follicles into two categories: the resting pool (consisting only of primordial follicles) and the growing pool (primary, secondary, pre-antral, and antral follicles). The PND18 *Amhr2-Cre/+;Notch2^flox/- ^*ovaries contained significantly fewer total follicles (40.6% of the control mice), and also significantly fewer follicles in the resting pool (only 16.7% of the control) (Table 3). A significantly reduced number of normal primordial follicles was seen in the *Amhr2-Cre/+;Notch2^flox/- ^*ovaries. The mutant mice had approximately nine-fold fewer primordial follicles containing a single oocyte than did their littermate controls (11.4% of control), and had 13 times more primordial follicles containing four or more oocytes than controls (Figure 4A). Regarding the growing pool, the *Amhr2-Cre/+;Notch2^flox/- ^*ovaries had significantly more primary and secondary follicles containing four or more oocytes, and also had more follicles of the primary, secondary, and pre-antral types containing two to three oocytes (Figure 4B-D). However, at PND18 the total number of follicles in the growing pool (primary, secondary, pre-antral, and antral) was not significantly different in the *Amhr2-Cre/+;Notch2^flox/- ^*and their littermate controls (Table 3).

**Table 2:** Oocyte quantification for *Amhr2-Cre/+;Notch2^flox/- ^*and control littermate mice.^a^

Genotype	Mice, n	Total number of oocytes^b^
*Amhr2-Cre/+;Notch2^flox^*/+	4	1001 ± 147
*Amhr2-Cre/+;Notch2^flox/-^*	4	1205 ± 233^c^

^a^Ovaries were harvested from mice at postnatal day 18. ^b^values are expressed as mean ± SD. ^c^*P *< 0.05.

**Table 3:** Follicle quantification for *Amhr2-Cre/+;Notch2^flox/- ^*and control littermate mice.

Genotype	Mice, n	Total number of follicles^a^	Resting pool^a, b^	Growing pool^a, c^
*Amhr2-Cre/+;Notch2^flox^*/+	4	3216 ± 494.8	2498 ± 290	718 ± 223
*Amhr2-Cre/+;Notch2^flox/-^*	4	1306 ± 415.1^d^	418 ± 195^e^	888 ± 223

^a^Values are expressed as mean ± SD. ^b^Primordial follicles. ^c^Primary, secondary, pre-antral, and antral follicles. ^d^*P *< 0.05, ^e ^< 0.005.

We used the PND18 oocyte quantification data to estimate the fraction of primordial germ-cell nests that failed to break down properly in *Amhr2-Cre/+;Notch2^flox/- ^*mice. We combined the oocyte numbers from all follicle types (primordial to antral), and assessed in both control littermate and *Amhr2-Cre/+;Notch2^flox/- ^*mice (*n *= 4 for each group) whether the follicles contained a single oocyte, or more than one oocyte (multi-oocyte follicle). In the control mice, 96.9% of the follicles contained a single oocyte, and the remaining 3.1% (399/12865) contained more than one oocyte. In the *Amhr2-Cre/+;Notch2^flox/- ^*mutants, 60.3% of the follicles contained a single oocyte, and the remaining 39.7% (2074/5223) contained more than one oocyte. Assuming that all multi-oocyte follicles resulted from a failure of primordial germ-cell-nest breakdown, these data suggest that approximately 40% of the primordial germ-cell nests in the *Amhr2-Cre/+;Notch2^flox/- ^*mice failed to break down and assemble into follicles with a single oocyte each.

### *Notch2 *gene function in granulosa cells is required for non-cell autonomous oocyte apoptosis

**Figure 5:**
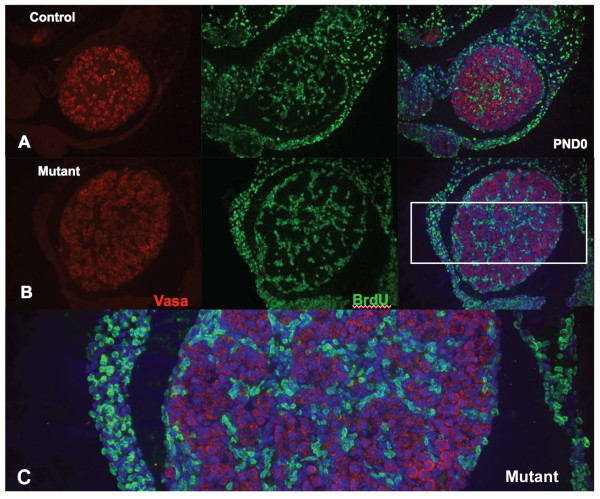
**Absence of extended oocyte proliferation in embryonic *Amhr2-Cre/+;Notch2^flox/- ^*ovaries**. Pregnant mice were injected with 5-bromo-2'-deoxyuridine (BrdU) twice daily between E16.5 through E18.5 (a total of six injections). Ovaries were isolated from *Amhr2-Cre/+;Notch2^flox/- ^*and control littermate progeny on the day of birth (postnatal day 0; PND0), and sections were stained with anti-Vasa (red) antibodies to label oocytes, and with anti-BrdU (green) antibodies to identify proliferating cells. Sections were also counterstained with DAPI (blue) to stain cell nuclei. As shown in the merged figure on the right, at PND0, no oocyte in either (**A**) littermate control or (**B**) mutant ovaries had incorporated BrdU as a result of these injections. (**C**) Higher magnification view of the mutant ovary (boxed region in **B**).

**Figure 6:**
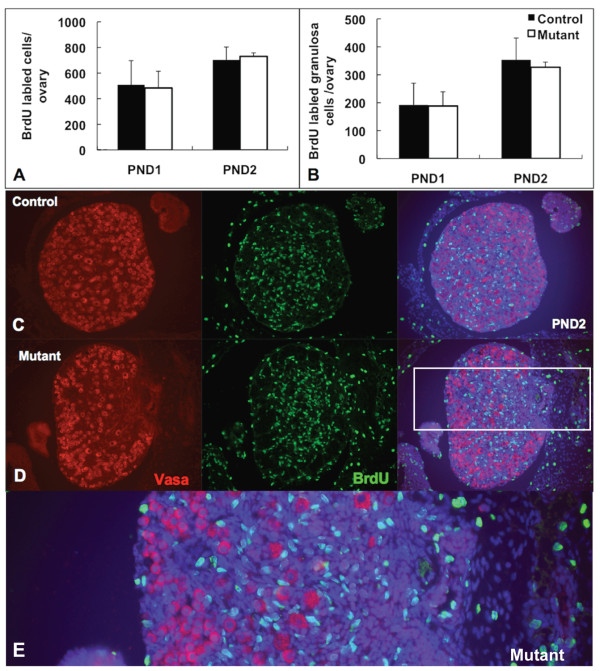
**Absence of extended cell proliferation in neonatal *Amhr2-Cre/+;Notch2^flox/- ^*ovaries**. (**A, B**) Quantification of (**A**) total 5-bromo-2'-deoxyuridine (BrdU)-positive cells and (**B**) BrdU-positive pre-granulosa/granulosa cells (defined as a BrdU-positive somatic cell adjacent to a Vasa-positive oocyte). Ovaries were harvested from *Amhr2-Cre/+;Notch2^flox/- ^*and control littermate mice after a 2-hour BrdU pulse administration on postnatal day (PND)1 or PND2. Data are presented as the mean ± SD of four ovaries from four individual mutant or control mice. There were no significant differences in the numbers of either (**A**) total proliferating cells or (**B**) proliferating pre-granulosa/granulosa cells between the control and the *Amhr2-Cre/+;Notch2^flox/- ^*ovaries. (**C**, **D**) Representative micrographs showing BrdU incorporation from (**C**) control littermate and (**D**) *Amhr2-Cre/+;Notch2^flox/- ^*ovaries at PND1. No oocyte in either (**C**) the control or (**D**) mutant ovary incorporated BrdU. BrdU-positive cells are in green; oocytes are in red (Vasa-positive), with nuclear DAPI staining in blue. (**E**) Higher magnification view of the mutant ovary (boxed region in **D**).

To address the mechanisms responsible for the 20% increase in oocyte numbers in *Amhr2-Cre/+;Notch2^flox/- ^*mice, we assessed cell proliferation (by 5-bromo-2'-deoxyuridine (BrdU) incorporation) and apoptotic cell death (by terminal deoxynucleotidyl transferase-mediated deoxyuridine triphosphate (TUNEL) assay) in ovaries of *Amhr2-Cre/+;Notch2^flox/- ^*and control littermate mice. In wild-type mouse embryos, oogonia cease mitosis and enter meiosis at approximately embryonic day 13.5. We assessed whether oocytes were still proliferating in *Amhr2-Cre/+;Notch2^flox/- ^*embryos during the period E16.5 to E18.5, using BrdU injection into timed pregnant females. Ovaries from the embryos of the BrdU-injected females were isolated and sectioned, and oocytes were identified by immunofluorescence staining with an antibody to the cytoplasmic germ-cell marker Vasa. We identified no oocytes throughout the period E16.5 to E18.5, in either *Amhr2-Cre/+;Notch2^flox/- ^*embryos or their littermate controls, which were double-stained with the anti-Vasa and anti-BrdU antibodies (Figure 5, and data not shown). We next examined cell proliferation in the ovaries of neonatal mice at PND1 and PND2 by BrdU injection at these time points. Quantifying either all BrdU-positive cells (Figure 6A) or BrdU-positive pre-granulosa/granulosa cells (which we defined as a BrdU-positive cell directly adjacent to a Vasa-positive oocyte) (Figure 6B), we detected no significant differences in the numbers of proliferating cells between the control and the *Amhr2-Cre/+;Notch2^flox/- ^*ovaries at either PND1 or PND2. Similarly to the embryonic BrdU exposure (Figure 5), we did not find any oocytes that were double-stained with the anti-Vasa and anti-BrdU antibodies, in either *Amhr2-Cre/+;Notch2^flox/- ^*embryos or their littermate controls, at PND1 or PND2 (Figure 6, and data not shown). Therefore, it did not appear that excess or extended proliferation was the cause of the increase in oocyte numbers in *Amhr2-Cre/+;Notch2^flox/- ^*mice.

**Figure 7:**
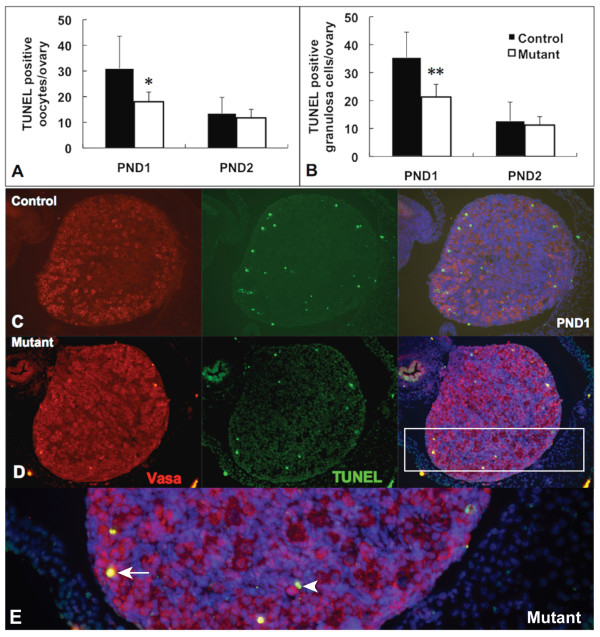
**Decreased apoptotic cell death in *Amhr2-Cre/+;Notch2^flox/- ^*ovaries**. (**A, B**) Quantification of terminal deoxynucleotidyl transferase-mediated deoxyuridine triphosphate (TUNEL)-positive (**A**) oocytes and (B) pre-granulosa/granulosa cells (defined as a TUNEL-positive somatic cell adjacent to a Vasa-positive oocyte). Data are presented as the mean ± SD of four ovaries from four individual mutant or control mice. At postnatal day (PND)1, there were significant differences in the numbers of both apoptotic (that is, TUNEL-positive) oocytes and pre-granulosa/granulosa cells between the control and the *Amhr2-Cre/+;Notch2^flox/- ^*ovaries. * *P *< 0.05; ** *P *< 0.01. (**C**, **D**) Representative micrographs showing TUNEL staining from (**C**) control littermate and^/^(**D**) *Amhr2-Cre/+;Notch2^flox ^*ovaries at PND1. TUNEL-positive cells are in green; oocytes are in red (Vasa-positive), with nuclear DAPI staining in blue. (**E**) Higher magnification view of the mutant ovary (boxed region in **D**). Arrow: TUNEL-positive oocyte; arrowhead: two TUNEL-positive pre-granulosa/granulosa cells adjacent to a Vasa-positive oocyte.

We next assessed apoptotic programmed cell death using TUNEL (Figure 7). Counting either TUNEL-positive oocytes (defined both by double staining with the TUNEL reaction and anti-Vasa antibody, and by the distinct morphology of the oocyte nucleus) (Figure 7A) or TUNEL-positive pre-granulosa/granulosa cells (defined as a TUNEL-positive cell directly adjacent to a Vasa-positive oocyte) (Figure 7B), we detected significantly fewer apoptotic cells of either type in *Amhr2-Cre/+;Notch2^flox/- ^*mutant ovaries at PND1. By PND2, the numbers of apoptotic cells (both oocytes and pre-granulosa/granulosa cells) were not significantly different in *Amhr2-Cre/+;Notch2^flox/- ^*and control ovaries. These data support a model in which the increase in oocyte number in *Amhr2-Cre/+;Notch2^flox/- ^*mutant ovaries is caused by reduced oocyte apoptotic cell death during the early postnatal stages.

## Discussion

Using a tissue-specific, conditional gene-deletion strategy, we investigated the role of the *Notch2 *gene in the mouse ovary by generating a granulosa-cell-specific *Notch2 *gene deletion with the *Amhr2-Cre *driver. Our results showed that *Notch2 *deletion in granulosa cells resulted in reduced fertility in the conditional mutant female mice, accompanied by the formation of multi-oocyte follicles that became hemorrhagic as the mice aged. Formation of multi-oocyte follicles resulted from defects in breakdown of the primordial germ-cell nests. In the ovaries of the *Notch2 *conditional mutant females, oocyte numbers were increased as a result of decreased oocyte apoptosis, but the number of normal primordial follicles containing a single oocyte was greatly decreased.

Previous studies have shown that the *Notch2 *gene is expressed at high levels in the somatic pre-granulosa [14,15] and granulosa [16] cell lineage of the mouse ovary. In addition, *ex vivo *culture of neonatal mouse ovaries with gamma-secretase inhibitors (which abrogate Notch signaling) resulted in defects in granulosa-cell proliferation and primordial-follicle formation [14,15], whereas overexpression of the Notch2 intracellular domain in these *ex vivo *cultures promoted granulosa-cell proliferation and rescued the growth inhibition induced by the gamma-secretase inhibitors [15]. Our study is the first genetic loss-of-function study confirming that Notch receptor function plays an essential physiological role in mammalian oogenesis and ovary development. In the *Notch2 *conditional deletion model, multi-oocyte follicles arise from persistence of germ-cell nests and reduced germ-cell apoptosis, indicating a non-cell autonomous effect on oocyte survival. These data provide *in vivo *evidence for the crucial function of the *Notch2 *gene in breakdown of germ-cell nests, postnatal apoptosis of oocytes, and formation of primordial follicles.

Other genetic mouse models that develop multi-oocyte follicles have been reported. These include targeted null mutations for *Dmrt4 *[20], *Ahch/Dax1 *[21], *Gcnf *[22], *Ghr *[23], *Bmp15/Gdf9 *double mutants [24], *Foxc1 *[25], the Notch signaling modulator *Lfng *[26], and transgenic mice with gain of function for rat inhibin-α [27]. However, a common feature of these models is the formation of multi-oocyte follicles with only a few (usually two or three) oocytes in a single follicle, rather than the large numbers of oocytes seen in the multi-oocyte follicles of *Amhr2-Cre/+;Notch2^flox/- ^*mice. One exception is the generation of mice with multi-oocyte follicles by neonatal administration of various estrogenic compounds, which also results in formation of multi-oocyte follicles containing large numbers of oocytes [28-30]. In that system, formation of multi-oocyte follicles is dependent on estrogen-receptor signaling. Formation of multi-oocyte follicles in neonatal mice treated with the soybean-derived phytoestrogen genistein was dependent on the presence of a functional β estrogen receptor (ERβ) but not on the α estrogen receptor (ERα) [28]. Mice homozygous for a targeted mutation of the *Esr1 *gene, which encodes ERα, formed multi-oocyte follicles when treated as neonates with genistein. However, mice that were homozygous mutant for the *Esr2 *gene (which encodes ERβ) did not form multi-oocyte follicles under these conditions, showing that ERα function is required for formation of multi-oocyte follicles after neonatal genistein administration. More recent work suggested that, depending on which estrogenic compound is delivered to the neonatal mice, ERα may also be required for multi-oocyte follicle formation [29,31]. Estrogen-receptor signaling might also contribute to the hemorrhagic follicle phenotype exhibited by *Amhr2-Cre/+;Notch2^flox/- ^*mice. Mice mutant for ERα develop hemorrhagic follicles [32-34] similar to those of the *Notch2 *conditional mutant mice, and this phenotype is exacerbated in ERα-ERβ double mutants [33]. It will be extremely interesting to determine if estrogen-receptor-mediated signaling from either the ERα or ERβ receptor contributes to the mutant phenotypes seen in *Amhr2-Cre/+;Notch2^flox/- ^*mice.

Our results clearly show that Notch2 function in somatic granulosa cells is required for breakdown of germ-cell nests and formation of primordial follicles. However, the identity and cellular source of the Notch ligand interacting with the Notch2 receptor in granulosa cells is not known. The model we favor is that expression of the Jagged family ligands Jag1 and/or Jag2 in the oocyte is the source of the ligand signal. Communication between the oocyte and the surrounding somatic cells (which has been termed the oocyte-granulosa-cell regulatory loop) is essential for the coordinated development of both the germ-cell and somatic-cell lineages [35,36]. Gene expression analyses have indicated that only the two Jagged family ligands Jag1 and Jag2 are expressed at high levels in the oocyte; Delta family ligands are expressed only at low to undetectable levels in the developing ovary [14-16,37]. Recent work has shown that Jag1-Notch2 signaling is downstream of neurotrophin 4/5 and brain-derived neurotrophic factor (BDNF)-tyrosine-related kinase (TRK) receptor B signaling in the ovarian follicle [38]. Expression of the *Jag1 *gene and the Notch target genes *Hes1 *and *Hey2 *is reduced in the ovaries of *TrkB *homozygous mutants at PND7. However, the *TrkB *homozygous mutant mice do not exhibit multi-oocyte follicles, indicating that upstream neurotrophin-TRK receptor signaling cannot explain all aspects of the *Notch2 *granulosa-cell-specific deletion phenotype.

However, another possible source for the ligand signal is via Jag2 expression in the granulosa cells themselves. *In situ *hybridization analysis indicates that the *Jag2 *gene is expressed more strongly in granulosa cells than in the oocyte [16]. Disruption of *cis *inhibition [39] of Notch2-mediated signaling by expression of the ligand Jag2 in granulosa cells is an alternative model to explain the requirement for Notch2 function in somatic granulosa cells. Determining where the relevant ligand signal is coming from (that is, oocytes or granulosa cells) by conditional gene-deletion experiments will be essential to obtain mechanistic understanding of how breakdown of germ-cell nests, postnatal apoptosis of oocytes, and formation of primordial follicles are regulated in wild-type mice, and how these processes are disrupted in the *Notch2 *conditional deletion model.

## Conclusions

We have shown that *Notch2*-mediated signaling in the somatic-cell lineage of the mouse ovary is essential for regulating breakdown of germ-cell nests and formation of primordial follicles. Female mice with conditional deletion of the *Notch2 *gene in somatic granulosa cells of the ovary exhibited reduced fertility, accompanied by the formation of multi-oocyte follicles. Formation of multi-oocyte follicles resulted from defects in breakdown of the primordial germ-cell nests. Ovaries of the *Notch2 *conditional mutant mice had increased numbers of oocytes, but decreased numbers of primordial follicles. Oocyte numbers in the *Notch2 *conditional mutants were increased not by excess or extended cellular proliferation, but as a result of decreased oocyte apoptosis. This model provides a new resource for studying the developmental and physiological role of Notch signaling during mammalian reproductive biology.

## Methods

### Ethics statement

All animal experiments were carried out with strict adherence to the National Institutes of Health (NIH) Guidelines for animal care and safety, and were approved by the Animal Care and Use Committee of the Jackson Laboratory (where the experiments were performed).

### Generation of granulosa-cell-specific *Notch2 *cKO mice

The *Amhr2-Cre *knock-in allele [19] was used to drive Cre recombinase expression in granulosa cells of the ovary. The *Amhr2-Cre *allele is expressed by E11.5 in both male and female urogenital ridges, and by E12.5, expression is localized to somatic cells of the gonads and mesenchyme cells of the Müllerian ducts [19]. At E17.5, *Amhr2-Cre *expression is present in almost all somatic cells of the ovary [40]. Construction and utilization of the *Notch2 *conditional allele (*Notch2^flox^*) and *Notch2 *null allele (*Notch2^del3^*) have been described previously [17,41]. To generate mice with targeted disruption of the *Notch2 *gene in ovarian granulosa cells, mice heterozygous for the *Notch2 *null allele and the *Amhr2-Cre *knock-in allele were crossed with mice homozygous for the *Notch2^flox ^*allele. Mice were genotyped by PCR. The *Notch2 *null allele was detected using the primers. Notch2 forward and Notch2 reverse (Table 4), and the *Notch2^flox ^*allele was detected using the primers Flox forward and Notch2 reverse (Table 4).

**Table 4:** Primers used for detection of Notch alleles.

*Primer*	*Sequence (5'→3')*
Notch2 forward	GCTCAGCTAGAGTGTTGTTCTTG
Notch 2 forward	ATAACGCTAAACGTGCACTGGAG

Flox forward	TAGGAAGCAGCTCAGCTCACAG

### Fertility test

To assess fertility, 7-week-old *Amhr2-Cre/+;Notch2^flox/- ^*and littermate control females (either *Notch2^flox/-^*, *Notch2^flox/+^*, or *Amhr2-Cre/+;Notch2^flox/+^*) were paired with a single male C57BL6/J mouse 7 to 8 weeks old. Cages were monitored daily, and the numbers of litters and litter sizes were recorded for a 6-month period.

### Histology and immunohistochemistry

Ovaries were removed, fixed overnight in Bouin's fixative for hematoxylin and eosin or Periodic-Acid-Schiff (PAS) staining, or at 4°C in 4% paraformaldehyde for immunohistochemistry. Fixed ovaries were embedded in paraffin wax, and cut into sections 6 μm thick. Sections used for immunohistochemistry were washed with phosphate-buffered saline and boiled for 10 minutes in 10 μmol/l sodium citrate (pH 6.0) for antigen retrieval. Sections were then incubated overnight at 4°C with primary antibodies (1:100 dilution) directed against: BrdU (BD Pharmingen, San Jose, CA, USA), Vasa (Ddx4) (Abcam, Cambridge, MA, USA), AMH (MIS, Santa Cruz, Dallas, TX, USA), and laminin (Sigma, St. Louis, MO, USA). After washing, sections were incubated with secondary antibodies, either Alexa fluor 488-conjugated or 546-conjugated donkey anti-mouse, anti-goat, or anti-rabbit (1:200; Invitrogen, Carlsbad, CA, USA), for 2 hours at room temperature, washed, and developed. Negative controls were prepared by omitting the primary antibody, and no staining above the background was detected. Sections from at least three mice of each genotype were processed for comparison of immunostaining, and immunohistochemical studies were repeated three to five times to ensure reproducibility of results.

### Cell proliferation

To examine germ-line cell proliferation, timed pregnant female mice were injected with a BrdU solution (Sigma, 10 mg/ml in 50:50 PBS/DMSO; final dose, 3 mg per mouse), twice daily (at 8-hour intervals) between E16.5 and E18.5. Following euthanasia, the ovaries were fixed and embedded in paraffin wax for sectioning. To study cell proliferation in mice on PND1 and PND2, female mice were injected intraperitoneally with BrdU solution at a final dose of 100 mg/kg (normal body weight approximately 2 g, hence 20 μl each). After 2 hours, the ovaries were harvested. Tissues were embedded in paraffin wax, and serial sections were cut at 6 μm. Sections were dewaxed and rehydrated, and stained with anti-BrdU and anti-Vasa antibodies. The total number of BrdU-positive cells in every 15th section was determined. Granulosa cells were defined as cells adjacent to the Vasa-positive germline cells.

### Morphological classification and quantification of follicles and oocyte counts

Ovaries of PND18 mice were sectioned at 6 μm and stained with PAS. The follicles in each ovary were counted serially in every third section through the entire ovary. Only healthy, non-atretic follicles with visible oocyte nuclei were scored. Follicles were classified as primordial, primary, secondary, pre-antral, or antral. Primordial follicles had a compact oocyte surrounded by a single layer of flattened granulosa cells; primary follicles had an enlarged oocyte surrounded by a single layer of cuboidal granulosa cells; secondary follicles had an enlarged oocyte surrounded by two layers of granulosa cells; and pre-antral follicles had an enlarged oocyte surrounded by three or four layers of granulosa cells. Any follicles with antral spaces were considered antral follicles. Total oocyte numbers at PND18 were counted serially in every 15th section through the entire ovary by counting every oocyte in the section. Total oocyte numbers at PND1 and 2 were counted serially in every 15th section through the entire ovary by counting every oocyte with positive Vasa and DAPI staining in the section.

### Terminal deoxynucleotidyl transferase-mediated deoxyuridine triphosphate nick end-labeling

For terminal deoxynucleotidyl transferase-mediated deoxyuridine triphosphate nick end-labeling (TUNEL) assay, ovaries were collected on PND1 and PND2, and fixed in cold 4% paraformaldehyde for 1 hour. Tissues were embedded in paraffin wax, and serially sectioned at 6 μm. Detection of apoptotic cells was carried out using a TUNEL apoptosis detection kit (Roche Applied Science, Indianapolis, IN, US), with co-staining using an anti-Vasa antibody. The total number of apoptotic oocytes and granulosa cells in every 15th section was counted.

### Statistical analysis

Data are presented as mean ± SD. A two-tailed *t*-test was performed to compare means between two groups, and one-way analysis of variance (ANOVA) was performed to compare means between multiple groups. *P*≤0.05 was considered significant.

## Abbreviations

BCNF: Brain-derived neurotrophic factor; DMSO: dimethyl sulfoxide; E: Embryonic day; ER: Estrogen receptor; PND: postnatal day; TRK: Tyrosine-related kinase; TUNEL: Terminal deoxynucleotidyl transferase-mediated deoxyuridine triphosphate

## Competing interests

The authors declare that they have no competing interests.

## Authors' contributions

JX and TG conceived and designed the study, analyzed data, and wrote the manuscript; and JX performed all the experiments. Both authors have read and approved the final manuscript.
